# Seasonal niche differentiation among closely related marine bacteria

**DOI:** 10.1038/s41396-021-01053-2

**Published:** 2021-07-20

**Authors:** Adrià Auladell, Albert Barberán, Ramiro Logares, Esther Garcés, Josep M. Gasol, Isabel Ferrera

**Affiliations:** 1 Departament de Biologia Marina i Oceanografia, Institut de Ciències del Mar, ICM-CSIC, Barcelona, Catalunya Spain; 2 Department of Environmental Science, University of Arizona, Tucson, AZ USA; 3 Center for Marine Ecosystems Research, School of Science, Edith Cowan University, Joondalup, WA Australia; 4 Centro Oceanográfico de Málaga, Instituto Español de Oceanografía, IEO-CSIC, Fuengirola, Málaga Spain

**Keywords:** Microbial ecology, Microbial ecology

## Abstract

Bacteria display dynamic abundance fluctuations over time in marine environments, where they play key biogeochemical roles. Here, we characterized the seasonal dynamics of marine bacteria in a coastal oligotrophic time series station, tested how similar the temporal niche of closely related taxa is, and what are the environmental parameters modulating their seasonal abundance patterns. We further explored how conserved the niche is at higher taxonomic levels. The community presented recurrent patterns of seasonality for 297 out of 6825 amplicon sequence variants (ASVs), which constituted almost half of the total relative abundance (47%). For certain genera, niche similarity decreased as nucleotide divergence in the 16S rRNA gene increased, a pattern compatible with the selection of similar taxa through environmental filtering. Additionally, we observed evidence of seasonal differentiation within various genera as seen by the distinct seasonal patterns of closely related taxa. At broader taxonomic levels, coherent seasonal trends did not exist at the class level, while the order and family ranks depended on the patterns that existed at the genus level. This study identifies the coexistence of closely related taxa for some bacterial groups and seasonal differentiation for others in a coastal marine environment subjected to a strong seasonality.

## Introduction

Marine microbial communities display dynamic abundance fluctuations over time, particularly in temperate coastal environments. Community structure changes on a daily, monthly, and annual scale due to the variation of bottom-up factors such as resource availability (including inorganic nutrients and dissolved organic carbon), top–down biotic interactions, and physical properties such as temperature, day length, or the presence of eddies and upwelling events [1]. Given that microbes are key players in the functioning of the biosphere, defining seasonality and understanding how taxa respond to changes in environmental conditions is crucial [2].

The establishment of microbial observatories across the globe in combination with the advances in sequencing methodologies has allowed the monitoring of microbial communities over time, from short- to long-term scales (see reviews by refs. [3, 4]). Various studies have shown remarkably repeatable seasonal patterns in the distribution and abundance of microbial taxa (i.e., [1, 5–8]), including those in the rare biosphere [9], and despite irregular environmental perturbations [10]. Further, investigating the dynamics of individual taxa —or finely resolved taxonomic units— on the short-term scale has revealed sharp turnover of communities mirroring environmental variability [11] and the relevance of interactions among microorganisms, influenced by the dynamics of phytoplankton blooms [12, 13]. On longer time scales, these high-resolution analyses have shown recurrent co-varying taxa (modules) regardless of the interannual variation in phytoplankton blooms [14] or a clear partitioning of modules of oligotrophs and copiotrophs over time [15]. Nevertheless, these patterns of module covariance can be lost under contrasting environmental conditions, as shown by a recent study [16]. In addition, the analysis of closely related populations of photoheterotrophic bacteria has shown that closely related amplicon sequence variants (ASVs) could represent distinct ecotypes occupying temporally different niches [17]. What is still missing is an in-depth study exploring the degree of niche similarity among closely related marine bacteria and how conserved the niche is at higher taxonomic levels.

Hutchinson proposed that an ‘n-dimensional hypervolume’ could define the niche of a species: a set of conditions under which an organism can survive and reproduce [18]. Together with abiotic parameters, biotic interactions such as mutualism, cross-feeding, and competition delineate the realized niche of taxa [19, 20]. The niche is determined both by homogeneous selection of traits to survive in a specific environment and heterogeneous selection for other traits to reduce competition that would facilitate coexistence [19]. In bacteria, genomic adaptations can come from horizontal gene transfer, gene polymorphisms, and other mutations mediated by these evolutionary selective processes. The analysis of these processes and how they impact the niche distribution is limited by the taxonomic resolution of the methodology used. Metagenomics has shown how multiple *Prochlorococcus* subpopulations with a distinctive set of flexible genes can temporally coexist [19], and has also uncovered a large amount of diversity within the SAR11 clade [20]. Although metagenomic data provide highly resolved taxonomic information, the technique is financially and computationally costly, which complicates scaling the analysis of these processes to the full community [21]. On the contrary, 16S rRNA gene amplicon sequencing is a cheap and efficient approach for broad community analyses. The limitation of this technique is however the genetic resolution of the 16S rRNA gene hypervariable regions, in the range of species delineation, yet easily allowing the genus differentiation [22, 23]. Coupled with time series studies of marine microbial observatories, this approach can thus inform on whether ecological distributions are shared within organisms at the sub-genus level (therein ‘closely related taxa’) [24]. Furthermore, it allows to extend this comparison to broader taxonomical groups and obtain insights into the ‘phylogenetic scale’ at which ecology presents coherence [25–27].

Here we used a monthly sampled time-series spanning 11 years from a coastal marine observatory in the North-Western Mediterranean Sea to explore the long-term seasonal trends in bacterioplankton communities. First, we evaluated how similar the temporal niche is between ASVs within the same genus, and later extended the comparison to broader taxonomic levels in order to answer the following questions: (1) how many ASVs are seasonal and what is the temporal distribution of distinct taxonomic groups, (2) how similar the niche among closely related ASVs within different marine genera is and what are the environmental parameters modulating their distinct ecological responses, and (3) how conserved the realized niche is as we go from genus to higher taxonomic levels (i.e., family, order, and class).

## Material and methods

### Location and sample collection

Samples were collected from the Blanes Bay Microbial Observatory (BBMO), a station located in the NW Mediterranean Sea about 1 km offshore over a water column of 20 m depth (41°40′N, 2°48′E) [28]. Sampling was conducted monthly over 11 years (January 2003 to December 2013). Water temperature and salinity were measured in situ with a conductivity, temperature, and depth probe, and light penetration was estimated using a Secchi disk. Surface seawater was pre-filtered through a 200 μm nylon mesh, transported to the laboratory under dim light in 20 L plastic carboys, and processed within 2 h. Chlorophyll *a* concentration was measured on GF/F filters extracted with acetone and processed by fluorometry [29]. The concentrations of inorganic nutrients (NO_3_^-^, NO_2_^-^, NH_4_^+^, PO_4_^3-^, SiO_2_) were determined spectrophotometrically using an Alliance Evolution II autoanalyzer [30]. The abundances of picocyanobacteria, heterotrophic bacteria, and photosynthetic pico- and nanoeukaryotes were determined by flow cytometry as described elsewhere [31]. Additionally, the abundance of photosynthetic and heterotrophic flagellates of different size ranges was measured by epifluorescence microscopy on 0.6 µm polycarbonate filters stained with 4′,6-diamidino-2-phenylindole. Microbial biomass was collected by filtering about 4 L of seawater using a peristaltic pump sequentially through a 20 μm nylon mesh (to remove large eukaryotes), a 3 μm pore-size 47 mm polycarbonate filter, and a 0.2 μm pore-size Sterivex unit (Millipore).

### DNA extraction, PCR amplification, and sequencing

DNA was extracted from the Sterivex unit (0.2 to 3 µm fraction of bacterioplankton) as described in ref. [32], purified, and concentrated in an Amicon 100 (Millipore) and quantified in a NanoDrop-1000 spectrophotometer (Thermo Scientific). DNA was stored at −80 °C and an aliquot from each sample was used for sequencing using a MiSeq sequencer (2 × 250 bp, Illumina) at the Research and Testing Laboratory (Lubbock, TX, USA; http://rtlgenomics.com/). Primers 341 F (5′-CCTACGGGNGGCWGCAG-3′) [33] and 806RB (5′-GGACTACNVGGGTWTCTAAT-3′) [34] were used to amplify the V3–V4 regions of the 16S rRNA gene. A total of 131 samples were successfully sequenced and used in subsequent analyses.

### Sequence processing

*DADA2* v1.12 was used to differentiate the partial 16S rRNA gene amplicon sequence variants (ASVs) and to remove chimeras [35]. Previously, spurious sequences and primers were trimmed using *cutadapt* v.1.16 [36]. Taxonomic assignment of the ASVs was performed with IDTAXA from *DECIPHER* v2.14 package [37] against the Genome Taxonomy Database (GTDB) r89 [38]. The GTDB has the advantage that incorporates new data from metagenomic assembled genomes (MAGs) and generates phylogenies based on 120 single-copy genes. Additionally, SILVA r138 taxonomy was used for nomenclature correspondence (see ASVs taxonomy in Supplementary Table 1 and the correspondence between databases in Supplementary Table 2) [39]. Compared to SILVA, GTDB allowed an increase in the assignation at the genus rank (14.6% more sequences) and the differentiation of new groups (e.g., D2472 genus within SAR86). Furthermore, the ASVs assigned to *Synechococcus* were checked against the Cyanorak database v2.1 [40] through 100% BLAST matches. ASVs classified as Mitochondria or Chloroplast were removed. ASV sequences were also clustered into OTUs (Operational Taxonomic Units) at 99% identity —a typical threshold for the delineation of OTUs in microbiome studies— for comparison purposes. Clustering was performed by calculating the nucleotide sequence distance matrix using the *DECIPHER* package. This matrix was also used to calculate the nucleotide divergence among ASVs.

### Community data analyses

We performed all analyses with the R v3.5 language [41]. For data processing we used the *phyloseq* v1.26 and *tidyverse* v1.3 packages [42, 43], and *ggplot2* v3.2 for visualization [44]. We defined abundant taxa as those above or equal to 1% relative abundance in at least one sample [45]. An ASV always below that cutoff was considered permanently rare. For both abundance groups, we defined three ASV categories based on occurrence: broad (≥75% occurrence), intermediate (>10% and <75% samples), and narrow (≤10% samples) distribution, as termed in ref. [14]. Abundant ASVs were further tested as Conditionally Rare Taxa (CRT), taxa typically in low abundance that occasionally become prevalent (bimodality = 0.9, relative abundance ≥1%) [46].

To estimate alpha diversity and beta diversity we used the *breakaway* v4.6 and *divnet* v0.34 packages, respectively [47, 48]. These approaches avoid common pitfalls from applying classical ecology indexes (i.e., Chao1, Shannon) to microbiome data, such as the influence of sequencing depth and data compositionality.

### Seasonality data analysis

To test whether each of the ASVs displayed seasonality —that is, recurrent changes over time— we used the Lomb Scargle Periodogram (LSP) as implemented in the *lomb* package v1.2 [49]. The method has previously been used for testing the seasonality of marine microbial communities [10]. The LSP determines the spectrum of frequencies composing the dataset. Afterwards, significance is tested through data randomizations (*q* ≤ 0.05, False Discovery Rate (FDR) correction). For each ASV, we obtain the density distribution for each of the periods and the peak normalized power (PN). The distribution shows which is the most recurrent period and the PN value measures the strength of this period. We considered the results as seasonal only if PN was above 10 and *q* ≤ 0.05, as in ref. [10]. The non-seasonal fraction is thus comprised of (1) truly non-seasonal ASVs, and (2) seasonal ASVs with no recurrent signal detected likely due to a limitation in our sequencing depth. In addition to the ASV level, we evaluated the seasonality at the class, order, family, and genus ranks. For a specific rank group (e.g., class Alphaproteobacteria), 80% of the ASVs were randomly chosen, aggregated, and the LSP was calculated (using 300 iterations). Out of the 29 classes present in the dataset, only the Alphaproteobacteria, Gammaproteobacteria, and Bacteroidia could be evaluated since these were the classes that presented more than one order, family, and genus ranks with at least 10 ASVs.

Further, we tested how the ASVs clustered based on the seasonal abundance patterns. First, we checked the number of possible clusters through the gap statistic from the *cluster* v2.1 package, since the expected number of clusters is unknown beforehand [50]. Afterwards, we clustered the data through hierarchical clustering. To visually compare the trend of the various seasonal ASVs, each one was fitted through a generalized additive model (GAM) using the *mgcv* v1.8 package [51]. The Centered Logarithm Ratio values (CLR, adding a pseudocount of 1) were fitted along the day of the year, allowing a smoothing parameter with 12 knots (the maximum number of curves, being 12 for the number of months) [52].

### Analyses of niche preference and environmental drivers

To examine if taxa within a given genus covary and, therefore, could share a realized temporal niche, we used the *propr* v4.2 package [53]. This package avoids the common pitfalls of compositional data analyzing correlation-like measurements. The raw counts are transformed to ratios, usually between the abundance of the taxon of interest and the geometric mean of all taxa for a specific sample. Then, for all the ratios of taxa A and taxa B, we measure the proportionality of change, Rho, with similar properties to a correlation measurement (see ref. [54] for a detailed explanation). The results are then filtered with a final FDR estimate of 5%.  Within each genus, we compared the Rho value between pairs of ASVs —acting as a proxy of niche similarity— against the nucleotide divergence among ASVs to see whether there were trends in niche relatedness. A linear model was used to test which genera presented significant relationships (*p* ≤ 0.05) between nucleotide divergence and Rho. We analyzed the genera with at least 10 closely related ASVs (at a maximum of 5 nucleotide divergence), which resulted in a total of 8 genera (out of 581). For most of these groups, using the V3 and V4 hypervariable regions of the 16S rRNA gene, 5 nucleotide divergence equals to a median sequence identity of 98.8% between two pairs. This nucleotide distance is the threshold that we used for considering two ASVs as closely related.

Finally, we tested which measured environmental parameters drive the patterns among closely related taxa. From the suite of measured variables, we selected temperature, chlorophyll *a* concentration, inorganic nutrient concentrations, and the abundance of photosynthetic nanoflagellates (PNF) and heterotrophic nanoflagellates (HNF). This selection was based on the expected relevance in modulating the ASV response (bottom up and top–down processes) and also considering the number of missing values in the dataset. Multicollinearity between the parameters was tested using the *HH* v3.1 package, showing no collinearity [55]. To model each ASV across the different parameters, we used the *corncob* v0.1 package [56] (FDR ≤5%). Afterwards, a display of the results was created with the GAM approach.

### Reproducibility

All the code including the parameters used for each package is available in the following repository: https://github.com/adriaaulaICM/bbmo_niche_sea. Sequence data have been deposited in the European Nucleotide Archive under project number PRJEB38773.

## Results

### Environmental, ecological, and taxonomic context

Surface water temperature at Blanes Bay varied seasonally, with minimal mean values in February (12.6 °C) and maximal values in August (24.5 °C, Supplementary Fig. 1). Inorganic nutrients were higher during autumn and winter while chlorophyll *a* reached the highest values (ca. 1 mg m^−3^) during the winter–spring transition. For a detailed description of the seasonality at Blanes Bay, including these and other environmental parameters, see ref. [28].

**Fig. 1:**
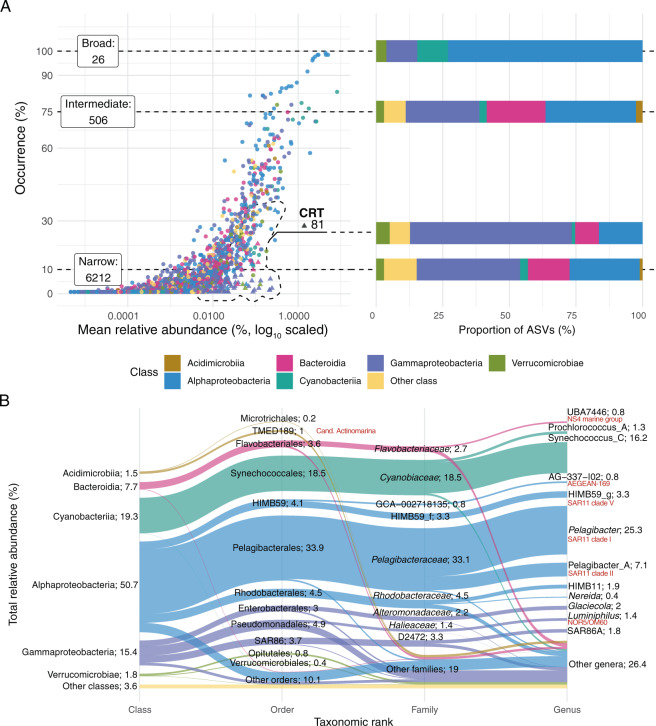
Community structure in the Blanes Bay Microbial Observatory. **A** Distribution of the different ASV types (broad, narrow or intermediate, and conditionally rare taxa, CRT). The *X* axis indicates the occurrence (% of samples) and the *Y* axis corresponds to the mean relative abundance (%) over the time series. Dotted lines delimitate the distributions (the numbers of ASVs of each type are displayed in the label) and connect to a box indicating the number of ASVs for each distribution and a bar plot colored by taxonomy at the class rank. CRT taxa are following a bimodal distribution and present ≥1% relative abundance in at least one sample. **B** Alluvial plot showing the total relative abundance distribution of Blanes Bay taxa across different taxonomic ranks (class, order, family, and genus). The height of the sections displays the relative abundance (indicated in the text; the total is 100%). The SILVA nomenclature is displayed in red next to the corresponding GTDB database nomenclature.

We detected a total of 6825 ASVs in the 11 years of monthly data. The ASV distribution was compared by occurrence (narrow: ≤10% occurrence; intermediate: >10% and <75%; and broad: ≥75%) and abundance (abundant or rare, i.e., <1% in all samples). Most of the ASVs (91%) displayed a narrow distribution (Fig. 1A, Table 1). Only 26 ASVs displayed a broad distribution, of which 3 always belonged to the rare fraction. Taxonomically, 19 of the broad ASVs belonged to the Alphaproteobacteria, mostly to the Pelagibacterales (13 ASVs) and HIMB59 (4 ASVs; former SAR11 clade V) orders. The 506 ASVs presenting an intermediate occurrence belonged to 20 different classes. The dominant classes for this category were the Alphaproteobacteria and Gammaproteobacteria (163 and 133 ASVs, respectively) followed by the Bacteroidia (106 ASVs), mostly by the Flavobacteriales order (91 ASVs; Fig. 1A). We also evaluated if rare ASVs occasionally became abundant (Conditionally Rare Taxa, CRT) and found a total of 81 ASVs. Gammaproteobacteria (48 ASVs) and Alphaproteobacteria (13) were the most common CRTs, while the rest belonged to the Verrucomicrobiae and Bacteroidia classes (Fig. 1B).

**Table 1:** Distribution, occurrence and relative abundance of the amplicon sequence variants (ASVs) in the Blanes Bay Microbial Observatory dataset. Distribution indicates the occurrence category: broad (≥75% samples), narrow (≤10% samples) and intermediate. The results are distributed between abundant (≥1% in at least one sample) and rare ASVs. Count ASVs stands for the number of ASVs within each category; Count CRT, the number of Conditionally Rare Taxa; seasonal ASVs, the count of seasonal ASVs (based in the Lomb Scargle test, *q* ≤ 0.05, PN ≥ 10); median occurrence, the % of samples in which the ASVs appear; Relative abundance, the total relative abundance of each category.

Distribution^*1*^	Count ASVs	Count CRT	Seasonal ASVs^*2*^	Median occurrence (%)	Relative abundance (%)
Abundant					
Broad	23	0	7	85.5	44.6
Intermediate	139	0	102	40.5	31.8
Narrow	11	0	0	7.6	0.2
CRT	81	81	4	3.1	5.0
Rare					
Broad	3	0	0	81.7	0.4
Intermediate	367	0	174	18.3	12.4
Narrow	6201	0	10	0.8	5.7

^*1*^Broad = in ≥75% of samples, Narrow = in ≤10% samples, Intermediate = in-between.

^*2*^Seasonality based in the Lomb Scargle test. PN ≥ 10, *q* ≤ 0.05.

In terms of alpha diversity, spring and summer displayed lower values than autumn and winter (*α* richness estimates = 197 vs 334 ASVs, respectively, *p* ≤ 0.01; Supplementary Fig. 2). Using January as intercept, we observed a significant decrease in richness in April (232 ASVs, *p* = 0.015) to regain higher values in October (316 ASVs, *p* = 0.87). Regarding community similarity (i.e., beta diversity), summer and winter displayed the maximum dissimilarity (*β* Bray Curtis estimate = 0.48, standard error = 0.036), while autumn and spring presented the lowest difference (*β* estimate = 0.21, standard error = 0.047; Supplementary Fig. 3), with similar ranges for all the other comparisons.

### ASV seasonality

A total of 297 ASVs out of 6825 were seasonal (Lomb Scargle Periodogram test *q* ≤ 0.05, PN ≥ 10) covering different ranges of occurrence and season maxima. These seasonal ASVs represented on average 47% of the read relative abundance, partitioned in 13% from ASVs exhibiting broad distribution, 34% of intermediate occurrence, and 0.1% of narrow presence. In our study, peak normalized power values —a statistic measuring how strong the recurrence is— ranged between 10 and 43.1. The highest values corresponded to ASVs with distributions that recurrently presented a peak in one particular season, often winter. ASV122, ASV55, and ASV131, belonging to the Acidimicrobiia, Bacteroidia, and Alphaproteobacteria classes, respectively, are examples of this pattern (Supplementary Fig. 4).

Within the seasonal ASVs, we differentiated 3 significantly different clusters (Supplementary Fig. 5). The first group, composed of 23 ASVs, includes most of the broadly distributed ASVs that peaked during summer and autumn. Taxonomically, this cluster was mostly composed of *Cyanobiaceae* and *Flavobacteriaceae* ASVs. The second cluster, of 30 ASVs, includes ASVs that peaked during winter and spring, mainly belonging to *Pelagibacteraceae*. Interestingly, this cluster includes the understudied group *Marinisoma* that displayed a winter trend in all its seasonal ASVs (5 out of 9 ASVs). Finally, the last cluster was composed of 244 ASVs without a clear seasonal pattern, likely due to their lower occurrence and relative abundance, without the dominance of a particular taxonomic group.

**Fig. 2:**
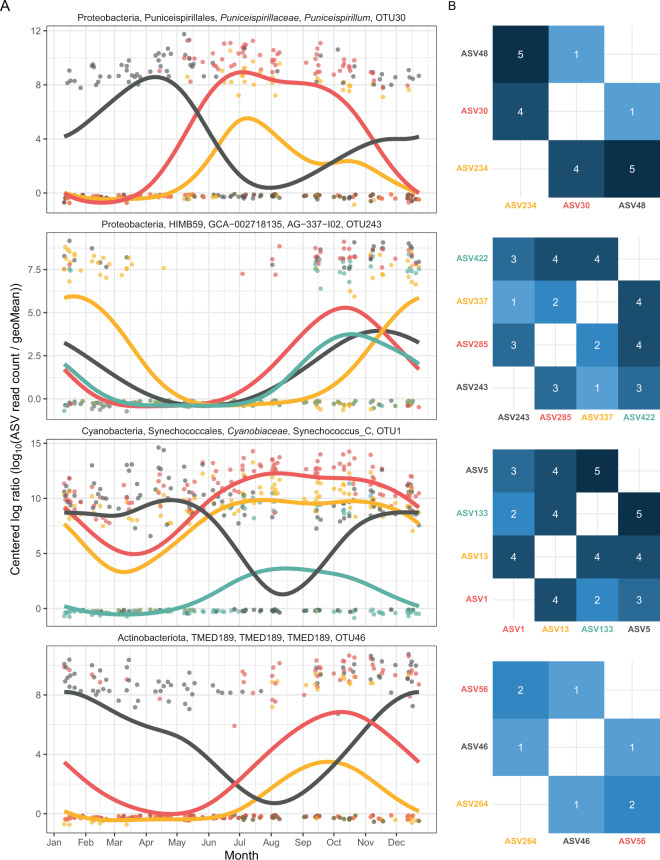
Examples of seasonal differentiation among closely related ASVs conforming the same OTU at 99% clustering. **A** Temporal abundance trends. The *X* axis presents the month and the *Y* axis presents the centered logarithm ratio abundance. A generalized additive model smooth is adjusted to the data points. **B** Heatmaps presenting the nucleotide divergence between each ASV pair (number of mismatches after alignment). Five nucleotide divergence equals to a median sequence identity of 98.8%.

In order to compare the seasonal trend of closely related taxa and investigate how frequent the presence of differentiated seasonal patterns at high sequence similarity is, we checked the ASVs that clustered at 99% similarity. We found 42 OTUs with ASVs presenting multiple ecological patterns. For example, *Pelagibacter* was represented by 20 different OTUs; 3 of them were composed only of seasonal ASVs, 6 OTUs contained both seasonal and non-seasonal ASVs, and 11 OTUs consisted only of non-seasonal ASVs. Similar trends were observed for other genera such as SAR86A and *Luminiphilus*. In general, we found that seasonal differentiation was not common, since only 20% of the OTUs contained ASVs with a clear difference. In total 8 ASVs displayed such behavior, that is, seasonal ASVs within 5 nucleotide mismatches presenting relative abundances with distinct temporal patterns (Fig. 2). Most of these patterns could be classified into either an almost complete temporal separation (e.g., ASV48 vs ASV30 within OTU30, affiliated to Puniceispirillales; Fig. 2) or a “restriction” of the temporal niche (one of the ASVs is only present in a specific month or season although the other is also present; e.g., ASV285 vs ASV337 within OTU243, affiliated to HIMB59). In fact, seven out of these 8 ASVs displayed the latter pattern of seasonal restriction.

### Variability of niche preference within genera

Here we define the ecological niche of a given taxon as the set of environmental conditions that fluctuate in this marine temperate coastal environment and that allows the growth of the microorganism or its persistence. Cooccurrence and covariance point to a possible niche similarity or mutualism. In our analysis, centered at variability within a genus, our proxy to test for niche overlap among closely related taxa is the Rho measurement (proportional change between two taxa), which can be expressed as a function of the nucleotide divergence between two sequences. A decrease in Rho as nucleotide distance increases denotes that the two taxa decrease their covariance, behaving less similarly as they become more phylogenetically distinct.

**Fig. 3:**
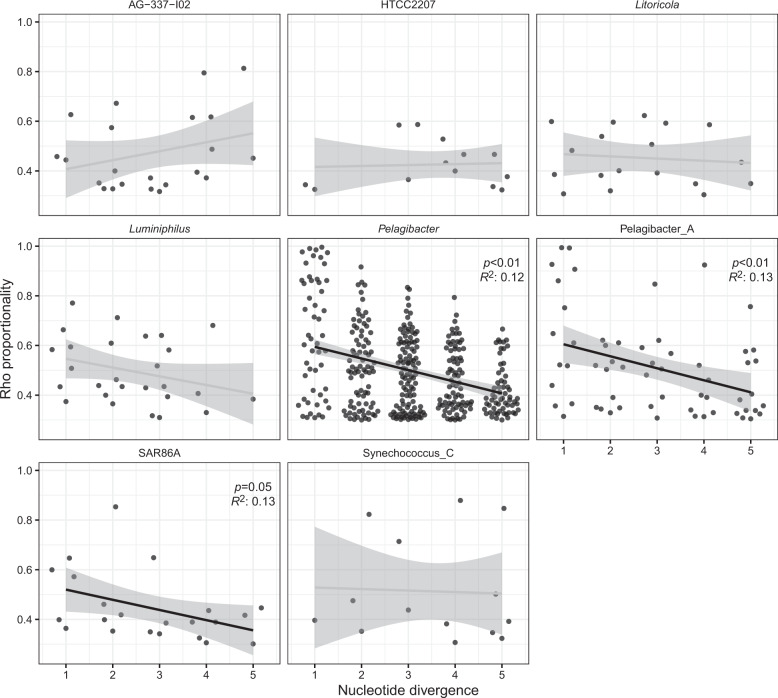
Relationship between the proportionality of change (Rho, *Y* axis) and the nucleotide divergence (mismatches after alignment, *X* axis). Only genera with more than 3 ASVs at less than 5 nucleotide divergences were evaluated. Gray and black lines represent the linear relationship between the two variables (black indicates statistical significance). The *p* value and the *R*^2^ are displayed for the significant regressions. See Supplementary Table 2 for the correspondence between GTDB and SILVA nomenclature.

Out of the 13 evaluated genera, we found that *Pelagibacter* (Alphaproteobacteria, SAR11 clade I), Pelagibacter_A (Alphaproteobacteria, SAR11 clade II), and less clearly SAR86A (a subclade of SAR86, Gammaproteobacteria) displayed a significant decrease in Rho proportionality when increasing nucleotide divergence (Fig. 3; Supplementary Table 3). The distributions within each genus were highly variable. *Pelagibacter* displayed the highest number of ASVs (60) and the variation in the Rho score was likewise the highest, between 0.3 and 0.996. Pelagibacter_A presented fewer ASVs (26) than *Pelagibacter* but a similar Rho distribution. SAR86A had a smaller amount of variation along with the nucleotide change, with a maximum Rho of 0.85. The *Synechococcus* genus (9 ASVs) displayed similarly high proportionality values at low and high nucleotide distances, not showing a decreasing trend. Merging all the non-significant genera, the values did not present a significant tendency (data not shown), suggesting that the decrease is specific to some groups.

### Environmental drivers of the observed niche differences within genera

**Fig. 4:**
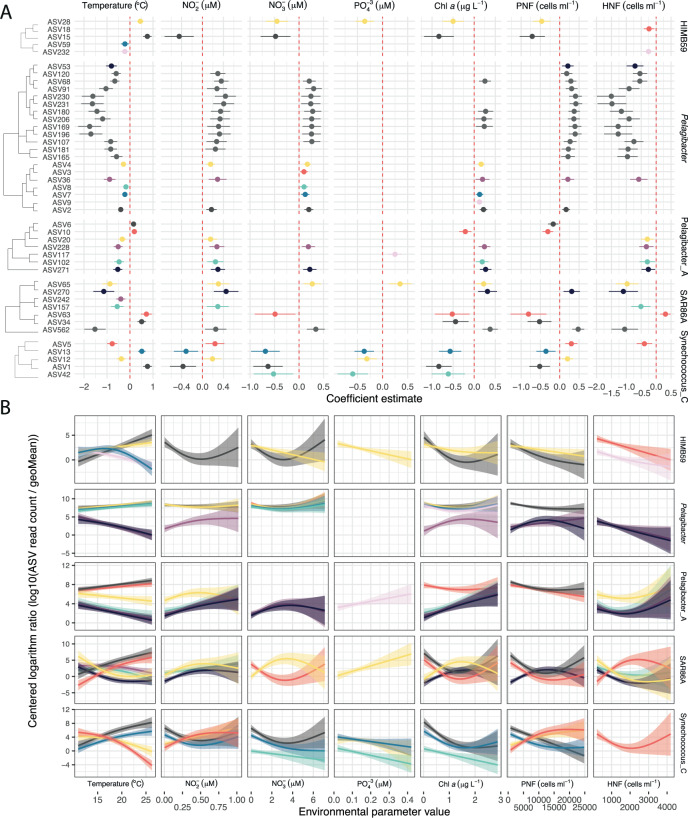
**A** Significant models among ASVs from HIMB59, Pelagibacter, Pelagibacter_A, SAR86 and Synechococcus genera (rows) and various environmental parameters (columns). The coefficient estimate indicates positive or negative responses to the parameter and is shown with a 95% confidence interval. The color corresponds to the different ASVs within a genus (only the top 8 more abundant ASVs are colored, the other ASVs are shown in gray). ASVs are ordered through a hierarchical clustering based on nucleotide divergence. **B** Generalized additive model fits between the ASV centered logarithm ratio abundances and the parameter value distribution for the significant ASVs in the upper plot. Panels and ASV colors shown as in **A**. PNF Phototrophic nanoflagellates, HNF Heterotrophic nanoflagellates.

Given the identified differences in the temporal niche among taxa, we evaluated how different environmental parameters influenced these distributions. For each ASV-parameter pair, we generated a model and the estimated coefficient indicating how the ASV responded (increase or decrease in abundance). A total of 245 out of the 603 response models were significant (FDR ≤ 0.05; Fig. 4, Supplementary Fig. 6). About two-thirds of the models were polynomial while the rest were linear. Temperature, nitrite, and nitrate concentrations were the parameters appearing most often, followed by the abundance of photosynthetic and heterotrophic nanoflagellates. The different bacterial genera responded divergently to the environmental parameters. *Pelagibacter*, AG-337-I02 (AEGEAN-169 marine group), D2472 (SAR86), and *Luminiphilus* had ASVs that responded cohesively to a given parameter, displaying the same response sign (Supplementary Fig. 6). Most of these bacterial genera showed a negative relative abundance response to temperature and a positive relationship with the concentration of inorganic nitrogen compounds. The exception to this trend was *Luminiphilus*, showing the opposite coefficient sign for all parameters. HIMB59 (former SAR11 clade V), Pelagibacter_A, SAR86A, and *Synechococcus* showed differences in the ecological patterns within each genus (Fig. 4A). Within SAR86A, two contrasting patterns could be observed; ASV34 and ASV63 (nucleotide divergence of 1; Supplementary Fig. 7) presented a positive relationship to temperature and a negative one to nitrate and chlorophyll *a* concentration, while ASV562, ASV270, ASV65, and ASV157 presented the opposite responses (these ASVs had nucleotide distances ranging from 1 to 9; Fig. 4A). In the case of *Synechococcus*, a similar trend was observed (ASV5 and ASV12 vs. ASV1 and ASV13, Fig. 4) but the phylogenetic distance does not hint to a possible explanation, as seen in the previous section (Fig. 3). Between ASV1 and ASV5 there was only a 3-nucleotide divergence (99.26% identity), but their seasonality was clearly different (Supplementary Fig. 8). We checked the *Synechococcus* ASVs taxonomy at a finer resolution using a picocyanobacterial-specific database, Cyanorak [40]. In particular, ASV5 presented a 100% identity match with strain PROS-9-1 belonging to Clade Ib, found in cold or temperate waters [57]. ASV1, on the other hand, resulted in a 100% match with members from multiple clades (Clades I, II, and III). In our long-term dataset, we found that the ASV5 peaks corresponded to the recurrent yet temporally restricted *Synechococcus* blooms observed during spring with flow cytometry (Supplementary Fig. 8). Pelagibacter_A also presented two specific responses with ASV6 and ASV10 (1 nucleotide divergence) responding similarly, in contrast to the other ASVs presenting a significant change within the genus (Fig. 4). Finally, the different ASVs belonging to HIMB59 (former SAR11 clade V) presented multiple responses (Fig. 4).

### Seasonality at broad taxonomical levels

**Fig. 5:**
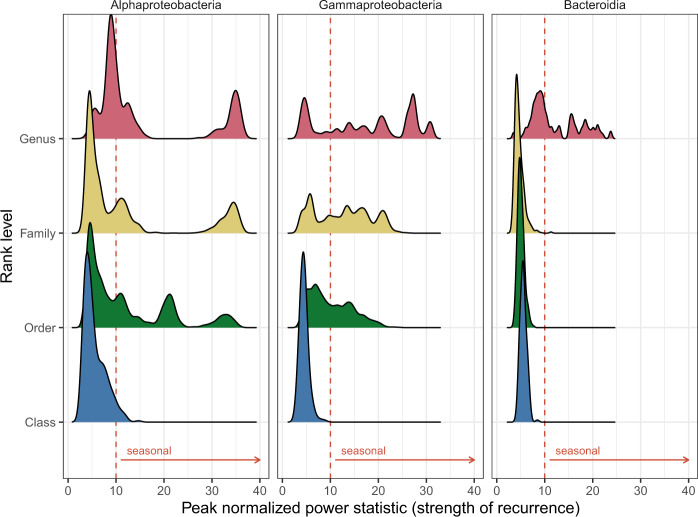
Density distribution of the peak normalized power statistic (as proxy for seasonality) for each rank level in the Alphaproteobacteria, Gammaproteobacteria, and Bacteroidia classes. The dotted lines indicate the used threshold for seasonality (*q* ≤ 0.05 and PN ≥ 10).

Having delineated how the ASVs behave seasonally and what are the drivers of these differences, we tested whether synchronized responses at higher taxonomic levels existed. When we analyzed the general distribution across ranks, we found that the class rank was mostly non-seasonal (98.9% peak normalized power-PN values, *p* ≤ 0.01, PN ≤ 10; Fig. 5). Both the order and family ranks displayed a similar distribution with ~50% of the results being seasonal, while this value increased up to ~60% at the genus rank. These distributions were different for each class; Alphaproteobacteria presented a clear bimodality while Gammaproteobacteria values were evenly distributed across the PN statistic (Fig. 5). By checking each level separately, the bulk Alphaproteobacteria class distribution (Supplementary Fig. 9, PN mean = 5.3) could be linked directly to that of the Pelagibacterales order, since this was the most abundant group (Supplementary Fig. 9B) and appeared as non-seasonal (PN mean = 5.7, Supplementary Fig. 9A). Observing the other prevalent orders (Rhodobacterales, Puniceispirillales –SAR116 clade– and HIMB59), the seasonality statistic was quite robust when randomly removing different ASVs (Supplementary Fig. 9). Puniceispirillales for example appeared mostly during summer. This observation was different for the Gammaproteobacteria orders (Supplementary Fig. 10A); the SAR86 and Pseudomonadales orders were close to the seasonality threshold resulting in half of the randomizations as non-seasonal. Moreover, for the Pseudomonadales order, we observed that it was composed of various families, each with different seasonality (Supplementary Fig. 10B). The Bacteroidia class only showed seasonality at the genus level for UBA7446, an uncultured genus within the family *Flavobacteriaceae* (Supplementary Fig. 11). Thus, the distributions at the order level were diametrically different, with Alphaproteobacteria including some seasonal orders, Gammaproteobacteria orders presenting a peak in the limit of seasonality, and all orders of Bacteroidia presenting a non-seasonal trend. Nevertheless, for most groups, the family and genus ranks presented similar seasonal trends to those displayed by the order to which they belonged.

## Discussion

We explored how marine bacterial communities are structured seasonally at fine taxonomical levels and whether the structure is maintained at higher ranks through long-term sampling and amplicon sequencing of the 16S rRNA gene in a temperate coastal environment. Specifically, we investigated how closely related ASVs responded to the environmental conditions that appeared recurrently at the coastal site. Overall, we found that around half of the total relative abundance of the community displayed seasonality at the ASV level. Within the genus level, we showed how niche similarity decreased with increasing nucleotide divergence for at least 3 genera. We then checked how various environmental parameters define the niche for the components of various genera. Finally, we analyzed how the patterns of seasonality aggregate at broader taxonomic ranks, proving that, in our dataset, the class level was non-seasonal and that the other ranks tested (i.e., order and family) presented a variety of trends.

As discussed above, the use of 16S rRNA gene amplicons has its limitations for the delineation of biological units [23]. The power of this genetic marker to resolve closely related taxa changes for different bacterial clades, but various studies have shown that species delineation is not always achievable by sequencing a region of this phylogenetic marker [22, 23]. Despite this limitation, amplicon marker gene sequencing still represents the fastest and most comprehensive approach for studying ecological patterns through identifying robust trends in large datasets. To stay on the conservative side in our interpretations, we set the within-genus level as the one for which we can assign patterns with certainty.

### Contrasting environmental conditions throughout the year

The environmental parameters displayed a clear seasonal pattern, with the highest rates of change between summer and winter, and the bacterial community mirrored these changes as observed in alpha diversity and community similarity (beta diversity). Patterns of alpha and beta diversity had been studied before at our study site but in much shorter surveys (1–2 years) [58, 59]. The analysis of eleven years of data unveiled that the highest differences in community structure also occurred between summer and winter, while the highest variability was found between spring and winter, which could be related to the recurrent phytoplankton blooms that occur during these periods, with differing intensity over the decade (see also the abundance of phototrophic nanoflagellates, PNF, in Supplementary Fig. 1) [60].

Patterns of community structure have been largely studied in different temperate coastal environments accurately describing yearly successions [3]. The community composition however can be driven by regional differences, such as the recurrence of phytoplankton blooms [61] or nutrient fluxes [16], modifying the bacterioplankton patterns from site to site. In the nearby long-term microbial station SOLA (Banyuls-sur-Mer, France), a seven-year seasonal study compared the bacterial, eukaryotic, and archaeal community through ASV delineation [10]. The number of ASVs in the bacterial community was similar to that observed here (6825 ASVs in this study vs 6242 at SOLA) and a similar community composition was observed, e.g., Pelagibacteraceae and Synechococcales dominated the communities at both sites. However, some differences were detected between our study and that of Lambert et al. [10]; a relevant group in Blanes Bay was the HIMB59 order, initially considered part of the SAR11 clade V [62, 63], which was absent from the SOLA station dataset [10, 64]. This result could either be related to primer biases or to differences in the taxonomic assignation. This group has been assigned a variety of names and phylogenetic positions; as an example, MAGs from the HIMB59 order were identical to the AEGEAN-169 marine group at the 16S rRNA gene comparison. This group, found in multiple surface and deep waters sites [7, 58], appears in the SILVA classification within the Rhodospirillales order. Martijn et al. [63] however concluded that the HIMB59 and other relevant MAGs conform a separate clade neither within the Pelagibacterales nor the Rhodospirillales, in agreement with the Genome Taxonomy Database assignation used here.

### Half of the total community is seasonal

Determining seasonality is not trivial, as it implies taking a binary decision for a trait that is likely continuous in a gradient rather than into two discrete states. In our analysis, we found a total of 297 seasonal ASVs (34% of the evaluated ASVs), which made up a total of 47% of the sequence relative abundance. This number of seasonal bacterial ASVs triplicates the results found by Lambert et al. [10] (89 ASVs), and the total relative abundance of seasonal bacteria was also higher in our study compared to that observed at the SOLA station (47% vs 31.3%). Since we followed the same statistical methodologies, the observed differences were somehow surprising. Differences in the length of the time series (7 years at SOLA vs 11 years at Blanes Bay) and the sampling scheme, with biweekly sampling at SOLA and monthly at Blanes Bay, could result to a certain degree in the observed disparities. Another explanation could derive from the presence of more irregular perturbations, such as river discharge in the Banyuls basin affecting the recurrence of the community through for example more variable salinity levels [65]. Further studies would be needed to find a possible explanation for these discrepancies.

The seasonal patterns observed in our time series varied among different taxonomic groups (Supplementary Fig. 5). Pelagibacter_A (SAR11 clade II) did not present seasonal ASVs; this result contrasts with what was observed in the Bermuda Atlantic Time series (BATS), where this group is present mostly during spring [66]. On the other hand, AG-337-I02 (order HIMB59) peaked during winter in Blanes, coinciding with what was observed at BATS (using SAR11 clade V as the group’s nomenclature). Nevertheless, the biogeochemical setting, physical forcing, and other environmental factors that could control the temporal dynamics at BATS [67] are quite different from those of the coastal NW Mediterranean. Besides, HIMB114 (SAR11 clade III) presented peak abundances during summer in Blanes, a result also observed in Banyuls-sur-Mer [64]. Our study thus complements the data existing from previous long-term datasets. A direct comparison of data from distinct sites would help understand these differences but this comparison is constrained by the different methodologies used (i.e., hypervariable region amplified or primer set used). When the sequencing of the complete 16S rRNA gene becomes a common practice, comparisons across microbial observatories will be easier to conduct [22].

### Niche similarity decreases with genetic distance in the 16S rRNA gene

Temporal distributions can inform on niche relatedness among closely related taxa. Specifically, cooccurrence and covariance could point to niche similarity. In this study, we found a clear trend between niche similarity and nucleotide divergence for *Pelagibacter*, Pelagibacter_A (i.e., SAR11 clade I and II), and less clearly for SAR86A. The pattern is consistent with environmental filtering, in which similar niches are occupied by closely related taxa sharing similar traits or adaptations, as seen previously for other taxonomic groups in environments such as lakes [64, 65]. Environmental filtering would include both abiotic (environmental filtering sensu stricto) and biotic factors such as ecological interactions [68, 69]. For most genera, however, there was no clear pattern. Since the 16S rRNA gene is very conserved, comparing niche similarity among ASVs could imply comparisons at broader level than that of strains. Each change in this marker gene can represent multiple changes at the genomic level, which could involve a change in niche distribution [23, 70]. In fact, even when merging the results for all the genera (excluding the SAR11 groups), there was no clear decrease in Rho with increasing nucleotide divergence. Nevertheless, as stated before, we observed a pattern for *Pelagibacter* and Pelagibacter_A. A possible reason for that observation is that these are the only groups presenting enough ASVs to result in a clear trend. Besides these two genera, others presenting a similar decrease pattern were SAR86A and *Luminiphilus*, which are the subsequent groups in number of ASVs per genera (22 and 26, respectively; Fig. 3). Another possible explanation is that the 16S rRNA gene could reflect in a greater way the genomic differences for *Pelagibacter* than for other groups, possibly due to the special evolutionary history of this group [66]. Both an increase in sequencing depth and an improvement of the resolution for the marker gene by sequencing a larger fragment could help to obtain a clearer picture [19].

When we checked how the individual ASVs responded to the measured environmental variables, we found two types of responses at the genus level: groups in which all the ASVs displayed a similar response, such as *Pelagibacter*, AG-337-I02 (AEGEAN-169), D2472 (SAR86) and *Luminiphilus*, and groups with ASVs presenting temporal differentiation, such as *Synechococcus* and SAR86A. The groups presenting the same patterns varied in their response; in the case of *Pelagibacter*, there was a clear distinction between the seasonal ASVs and the ones appearing all year round (e.g., in Fig. 4, see the two clusters in the *Pelagibacter* dendrogram). *Pelagibacter* therefore presented multiple variants with similar responses to the studied environmental changes [71]. On the other hand, different *Synechococcus* ASVs presented completely different adaptations — e.g., ASV1 and ASV5 — in an example of a clear niche switch by a previous ecotype differentiation. In the latter case, ASV1 presented multiple matches in the Cyanorak database, which exemplifies the problems with the limited power of the 16S rRNA gene V3–V4 regions to resolve species for certain groups [22]. This could reflect that there are many clades considered as the same ASV, which could explain that this variant dominates all year round. Summing up, these results illustrate the diversity of ecological trends within genera, which would have been hidden using sequence clustering methods.

### Lack of seasonality at the class level

It has been hypothesized that phylogenetic related taxa could share ecological traits and respond similarly to environmental changes [25, 26] but it is unclear whether bacteria from the same genus, family, order, or class phylogenetic ranks are ecologically cohesive [25]. These ecological traits could be determined by phylogenetic history, as seems to be the case of particle-attached vs free-living lifestyle [72, 73]. In the case of surface coastal waters, periodic changes in environmental conditions should promote recurrent niches. By randomly aggregating the ASVs at different ranks, broad patterns of abundance could emerge coming from cohesive seasonal responses. Our results were opposite to those observed in the English Channel, with the Alphaproteobacteria and Gammaproteobacteria classes presenting a high autocorrelation driven by a strong seasonal pattern [6, 74]. The higher annual temperature range in the English Channel could explain the observed differences compared with Blanes Bay, with less temperature variability. By facing a stronger environmental gradient, the whole community composition could consequentially change at a higher taxonomic rank. Bimodal distributions (seasonal and non-seasonal results) originate in groups containing ASVs that have strong seasonal trends and other non-seasonal ASVs, as is the case for Rhodobacterales and Pseudomonadales, copiotrophic groups occupying many different niches. *Rhodobacteraceae*, for example, includes ASVs with seasonal peaks in every season (Supplementary Fig. 5). Finally, the groups with all ASVs being seasonal could present more constrained optimal conditions of growth than those groups that appear randomly or all year-round. Examples of this behavior are the Puniceispirillales (SAR116 clade), a group harboring proteorhodopsin [75] for which most of the ASVs were seasonal and peaked during summer [75]. Metagenomic and genome-centric approaches as well as physiological experimentation with available isolates would help shedding light on the traits that determine the niche for these cohesive groups and the differences with the more diverse groups.

## Conclusions

The use of a long-term time series and fine-grained taxonomic resolution through the use of ASVs allowed to compare within-genus ecological distributions in a coastal site. Specifically, we could prove that for certain genera niche similarity decreased with 16S rRNA gene nucleotide divergence, indicating that more similar variants coexist. Our results thus point to environmental selection as an important process structuring the seasonal dynamics of the studied microbiota. Both abiotic conditions and biotic processes (e.g., competition and other interactions) would exert selection in the analyzed community. Additionally, through modeling of the differential abundance with a variety of environmental parameters, we unveiled the presence of different ecological patterns spanning different seasons. Finally, the analysis of different seasonality distributions for each phylogenetic rank indicated that the class rank was non-seasonal for the groups analyzed, being thus ecologically non-coherent. Contrarily, some groups at the family and genera ranks presented cohesive responses. Overall, this study sheds light on the niche specialization of relevant genera in marine coastal microbial communities.

## Supplementary information

Supplementary Figures 1-11

Supplementary table 1

Supplementary table 2

Supplementary table 3
