# Supplementary material for: Seasonal niche differentiation among closely related marine bacteria
Source: ISME J. 2021 Jul 20;16(1):178–89. doi: 10.1038/s41396-021-01053-2 (PMC8692485; doi:10.1038/s41396-021-01053-2)
Supplement: Supplementary file 3 — Supplementary table 2 [file 41396_2021_1053_MOESM3_ESM.pdf]

Genus (GTDB r89)	Order + family (GTDB r89)	Genus (SILVA r138)	Order + family (SILVA r138)	N. seasonal	Total tested ASVs	General information genus
**AG-337-I02**	HIMB59, GCA-002718135	-	Rhodospirillales, AEGEAN-169 marine group	12	20	Rhodospirillales order is broken in 4 different orders in GTDB. New studies and data have excluded HIMB59 as a new order outside Rhodospirillales (see *Martijn et al. 2018*).
**AG-422-B15**	Pelagibacterales, AG-422-B15	-	SAR11 clade, Clade IV	2	5	-
**D2472**	SAR86, D2472	-	-	3	12	SAR86 order presents 4 families in GTDB: D2472, SAR86, AG-339-G14 and TMED112. In this study we only found assignation for the first family.
**HIMB114**	Pelagibacterales, *Pelagibacteraceae*	-	SAR11 clade, Clade III	4	5	-
**HIMB59**	HIMB59, HIMB59	-	Rhodospirillales, AEGEAN-169 marine group	2	8	Similar observations to AG-337-I02.
**HTCC2207**	Pseudomonadales, Porticoccaceae	SAR92 clade	Cellvibrionales, Porticoccaceae	0	17	-
Litoricola	Pseudomonadales, *Litoricolaceae*	Litoricola	Oceanospirillales, *Litoricolaceae*	5	8	-
Luminiphilus	Pseudomonadales, *Halieaceae*	*Luminiphilus*	Cellvibrionales, *Halieaceae*	9	30	Some assignations included the group in OM60(NOR5) clade for SILVA.
Marinisoma	Marinisomatales, *Marinisomataceae*	-	-	5	8	Marinisomatota phyla. Not described so far.
**MS024-2A**	Flavobacteriales, *Flavobacteriaceae*	NS5 marine group	Flavobacteriales, *Flavobacteriaceae*	4	6	-
**OM182**	Pseudomonadales, *Pseudohongiellaceae*	*Pseudohongiella*	Oceanospirillales, *Pseudohongiellaceae*	7	15	Oceanospirillales order is included inside Pseudomonadales.
Pelagibacter	Pelagibacterales, *Pelagibacteraceae*	Clade Ia	SAR11 clade, Clade I	20	63	In some cases the clade was Ib or the assignation was unknown. In this case GTDB unifies instead of splitting as with other groups.
**Pelagibacter_A**	Pelagibacterales, *Pelagibacteraceae*	-	SAR11 clade, Clade II	0	27	-
Puniceispirillum	Puniceispirillales, *Puniceispirillaceae*	Cand. Puniceispirillum	Puniceispirillales, SAR116 clade	3	5	-
**SAR86A**	SAR86, D2472	-	-	11	26	-
**SCGC-AAA076-P13**	SAR86, D2472	-	-	4	9	-
**Synechococcus_C**	Synechococcales, *Cyanobiaceae*	*Synechococcus* CC9902	Synechococcales, *Cyanobiaceae*	4	9	*Synechococcus* presents 4 genera in GTDB.
**TMED189**	TMED189, TMED189	Cand. *Actinomarina*	Actinomarinales, *Actinomarinaceae*	4	7	*Actinomarina* assignation is not present in GTDB. Since the assignation comes from *Ghai et al. 2013* this will probably be corrected in further versions of the DB.
**UBA4421**	Pseudomonadales, HTCC2089	-	-	2	7	-
**UBA7446**	Flavobacteriales, *Flavobacteriaceae*	NS4 marine group	Flavobacteriales, *Flavobacteriaceae*	3	10	-
