# Supplementary material for: Seasonal niche differentiation among closely related marine bacteria
Source: ISME J. 2021 Jul 20;16(1):178–89. doi: 10.1038/s41396-021-01053-2 (PMC8692485; doi:10.1038/s41396-021-01053-2)
Supplement: Supplementary file 4 — Supplementary table 3 [file 41396_2021_1053_MOESM4_ESM.pdf]

Genus	*df*	logLik	AIC	BIC	deviance	*df* (residual)	*p*	R^2
Pelagibacter	2	171.5	–337.1	–325.2	9.1	380	<0.0001	0.126
SAR86A	2	15.2	–24.3	–21.0	0.3	20	0.052	0.135
Litoricola	2	14.3	–22.6	–19.9	0.2	16	0.683	–0.051
Pelagibacter_A	2	18.7	–31.5	–25.2	1.8	57	0.003	0.130
Synechococcus_C	2	2.6	0.8	2.7	0.6	12	0.89	–0.082
Luminiphilus	2	18.2	–30.4	–26.6	0.4	25	0.13	0.053
AG-337-I02	2	11.3	–16.6	–13.4	0.5	20	0.19	0.038
